# The Putative Endoglucanase PcGH61D from *Phanerochaete chrysosporium* Is a Metal-Dependent Oxidative Enzyme that Cleaves Cellulose

**DOI:** 10.1371/journal.pone.0027807

**Published:** 2011-11-23

**Authors:** Bjørge Westereng, Takuya Ishida, Gustav Vaaje-Kolstad, Miao Wu, Vincent G. H. Eijsink, Kiyohiko Igarashi, Masahiro Samejima, Jerry Ståhlberg, Svein J. Horn, Mats Sandgren

**Affiliations:** 1 Department of Chemistry, Biotechnology and Food Science, Norwegian University of Life Sciences, Ås, Norway; 2 Department of Biomaterials Sciences, Graduate School of Agricultural and Life Sciences, University of Tokyo, Tokyo, Japan; 3 Department of Molecular Biology, Swedish University of Agricultural Sciences, Uppsala, Sweden; University of South Florida College of Medicine, United States of America

## Abstract

Many fungi growing on plant biomass produce proteins currently classified as glycoside hydrolase family 61 (GH61), some of which are known to act synergistically with cellulases. In this study we show that *Pc*GH61D, the gene product of an open reading frame in the genome of *Phanerochaete chrysosporium,* is an enzyme that cleaves cellulose using a metal-dependent oxidative mechanism that leads to generation of aldonic acids. The activity of this enzyme and its beneficial effect on the efficiency of classical cellulases are stimulated by the presence of electron donors. Experiments with reduced cellulose confirmed the oxidative nature of the reaction catalyzed by *Pc*GH61D and indicated that the enzyme may be capable of penetrating into the substrate. Considering the abundance of GH61-encoding genes in fungi and genes encoding their functional bacterial homologues currently classified as carbohydrate binding modules family 33 (CBM33), this enzyme activity is likely to turn out as a major determinant of microbial biomass-degrading efficiency.

## Introduction

Traditionally, enzyme systems capable of degrading recalcitrant polysaccharides, such as cellulose and chitin, are thought to consist of endo-acting enzymes that are cutting randomly in the polysaccharide chain, and processive exo-acting enzymes (chito- or cellobiohydrolases) that degrade the polymers from chain ends [1]. These enzymes often contain one or more carbohydrate binding modules that may contribute to their activity by promoting substrate binding and perhaps even some sort of substrate disrupting action [2], [3]. Still, it remains difficult to understand how these glycoside hydrolases could gain access to a polysaccharide chain in its crystalline environment, and one may wonder about the existence of additional factors that would make the substrate more accessible [4]. Recent studies of bacterial proteins currently classified as family 33 Carbohydrate Binding Modules (CBM33) [5] have shown that the classical endo/exo scheme indeed may be too simple [6], [7]. CBM33 proteins have flat substrate-binding surfaces and are capable of cleaving polysaccharide chains in their crystalline contexts using an oxidative mechanism that depends on the presence of bivalent metal ions and an electron donor [7]. Most importantly, CBM33 activity can boost the activity of classical cellulases or chitinases towards certain recalcitrant forms of cellulose and chitin, respectively.

Biomass-degrading fungi produce a plethora of enzymes when growing on cellulose [8], [9], [10]. Among proteins upregulated during growth on cellulose are proteins that are currently classified as Glycoside Hydrolases family 61 (GH61) [11], [12]. It has been claimed that GH61 proteins exhibit weak endoglucanase activity [13], but structural [14] and recent functional [15] studies indicate that they are not classical glycoside hydrolases. Since GH61 proteins structurally resemble CBM33s [14] and since some of them are known to act synergistically with cellulases [15], it has been suggested that GH61 proteins may be the fungal analogues of CBM33's [16]. A recent study [17], showing that a combination of a GH61 from *Thermoascus aurantiacus* (*Ta*GH61A) and cellobiose dehydrogenase (CDH) acts synergistically with cellulases and yields oxidized products, may be taken to support this suggestion. So far, very little is known about these GH61 proteins and more experimental work is needed to unravel their functions.

Genes coding for products classified as GH61 are abundant in the genomes of biomass-degrading fungi. One of the most extreme examples is *Coprinopsis cinerea* the genome of which contains as many as 33 putative GH61-encoding genes [12]. According to current publicly available annotations [18], [19], the genome of the wood-degrading white-rot fungus *Phanerochaete chrysosporium* has at least 11 genes encoding a GH61 single domain protein or a GH61 fused to a carbohydrate-binding module (CBM). In a recent study on the *P. chrysosporium* secretome [10], it was shown that CDH and GH61 proteins are upregulated by xylan. In this study, we have identified a GH61 protein, hereafter referred to as *Pc*GH61D, the expression of which is also upregulated when the fungus is grown on xylan. We have cloned and overexpressed *Pc*GH61D in *Pichia pastoris* and we have used purified correctly processed recombinant protein to characterize its function. We show that *Pc*GH61D cleaves cellulose using an oxidative mechanism and, by doing so, acts synergistically with cellulases.

## Materials and Methods

### Materials

*Phanerochaete chrysosporium* strain K-3 [20] was used as the source for the target gene. *Escherichia coli* strain JM109 (Takara Bio, Otsu, Japan) was used as subcloning host and *Pichia pastoris* strain KM71H (Invitrogen, Carlsbad, CA) for heterologous production of recombinant *Pc*GH61D. The primers for subcloning and construction of expression vector were purchased from Proligo (Boulder, CO), and the primers for Quikchange were purchased from Invitrogen (Carlsbad, CA, USA). The deglycosylation enzyme, EndoH (endo-β-N-acetyl glucosaminidase, EC 3.2.1.96, from *Streptomyces plicatus*), was a kind gift by Genencor, a Danisco division (Palo Alto, CA, USA).

### Protein identification and cloning of cDNA encoding PcGH61D from P. chrysosporium

The identification of *Pc*GH61D protein was carried out as described in [10]. *P. chrysosporium* strain K-3 [20] was cultivated at 26.5°C using a Kremer and Wood's minimal medium [21] with 2% cellulose as carbon source (CF11; Whatman, Fairfield, NJ, USA), and with or without an addition of 0.2% (w/v) beech xylan (Sigma-Aldrich, St. Louis, MO, USA) as described in [10]. After 3 days of cultivation the mycelium was collected by filtrating the culture medium, and collected mycelium was frozen with liquid nitrogen. Proteins in the culture medium were analyzed using two-dimensional electrophoresis as described in [10]. The N-terminal sequences of interesting proteins was determined by sequencing the proteins on a protein sequencer from Applied Biosystems (model 491 cLC; Applied Biosystems, Foster City, CA, USA) using the protocol described in [22]. This procedure led to the identification of *Pc*GH61D. The synthesis of cDNA from *P. chrysosporium* culture was carried out using the same protocol as described in [23]. Total RNA was extracted from approximately 100 mg of mycelial powder of *P. chrysosporium* culture grown on cellulose media, using an E.Z.N.A Fungal RNA Kit (Omega Biotech, GA, USA). The first strand of the cDNA was synthesized using a Ready-To-Go™ T-primed First-strand cDNA synthesis Kit (GE Healthcare UK Ltd., Buckinghamshire, UK). Based on the annotated gene model ID 4691 (scaffold_7:1591640-1592893 in the *P. chrysosporium* v. 2.0 genome database at the JGI genome portal (http://genome.jgi-psf.org/Phchr1/Phchr1.home.html)), the nucleotide primers F1: 5′-CACTACACCTTCCCCGACTTCATTGAGCCTAGC-3′ and R1: 5′-TCCTTGCCAGACAGCGGGGCCAG-3′ were designed for PCR amplification of the cDNA sequence. The amplified fragment was ligated into pGEM-T Easy Vectors (Promega, Madison, WI, USA) and transformed into *E. coli* strain JM109. The fragment was sequenced using a Thermo Sequenase primer cycle sequencing kit (GE Healthcare) using a DNA sequencer model SQ5500E (Hitachi High Technologies, Tokyo, Japan), as described previously [23].

### Nucleotide sequence accession number

The nucleotide sequence of the cDNA encoding *Pc*GH61D has been submitted to DDBJ/EMBL/GeneBank databases with accession number: AB670125.

### Heterologous expression of recombinant PcGH61D in P. pastoris

The oligonucleotide primers F2: 5′-TTTGAATTCCACTACACCTTCCCCGACTTC-3′ and R2: 5′-TTTGCGGCCGCTATCCTTGCCAGACAGCG-3′, introducing *EcoRI* and *NotI* cleavage sites, respectively (underlined sequence) were designed to amplify a fragment of *Pc*GH61D cDNA sequence encoding the predicted mature enzyme (i.e. without secretion signal in the cDNA sequence). The PCR product was ligated into the *P. pastoris* pPICZα-A vector (Invitrogen, Carlsbad, CA) using the same restriction sites as in the PCR amplified *Pc*GH61D cDNA product. The resulting plasmid has an *EcoRI* restriction site that introduces additional amino acid residues, Glu-Phe, at the N-terminal of the expressed protein, as well as a STE13 protease site that may cause heterogeneous N-terminus of the protein. To substitute the STE13 site and the *EcoRI* restriction site in the plasmid with an enterokinase cleavage site, the pPICZα vector containing the *Pc*GH61D cDNA sequence was modified by means of the Quikchange® method (Agilent Technologies, Santa Clara, CA, USA), using the primers 5′-CTCTCGAGAAAAGAGATGATGACGACAGACACTACACCTTCCC -3′ and 5′-GGGAAGGTGTAGTGTCTGTCGTCATCATCTCTTTTCTCGAGAG-3′. In the resulting final construct, the sequence near the start of the mature product looks as follows (His1 in bold face; enterokinase recognition motif in italics, enterokinase cleavage site indicated by a slash): LEKR*DDDDR* / **H**YTF.

Transformation of *P. pastoris* was conducted as described in [24]. Prior to transformation of *P. pastoris*, approximately 5 µg of the plasmid was linearized using the restriction enzyme Bpu1102I (Takara Bio, Otsu, Japan). Electroporation and selection of transformants were carried out according to the EasySelect™ *P. pastoris* expression kit standard protocol (Invitrogen, Carlsbad, CA). The expression of recombinant protein was performed as described in [25]. After induction with 1% (w/v) methanol for 3 days, *P. pastoris* cells were removed from the culture medium by centrifugation of the culture for 15 min at 1500 x g.

### Deglycosylation, protease treatment and N-teminal sequencing of the expressed PcGH61D protein

One mL of the culture supernatant, from expression in *P. pastoris*, was taken for checking the effect of enzymatic processing mentioned below. After concentration using VIVAspin 500 (GE Healthcare UK Ltd., Buckinghamshire, UK), 2 µL of concentrated culture medium, estimated at approximately 1 mg/mL protein concentration (BioRad Protein assay with BSA as standard), was incubated with 10 ng of EndoH for 30 min in 20 mM citrate buffer pH 5.5 at 35°C. One EndoH treated protein sample was diluted to 20 µL and 50 mM Tris-HCl buffer pH 8.0 with 2 mM calcium chloride and 50 mM sodium chloride, and then further incubated with 1 ng of enterokinase, light-chain (New England Biolabs) at 25°C for 16 hours. The protein samples treated by only EndoH and both EndoH and enterokinase were subjected to SDS-PAGE followed by protein transfer to a polyvinylidene difluoride membrane (Millipore) using a Trans-Blot SD cell (Biorad). N-terminal amino acid sequences of the protein was determined using the same method as described above.

### Purification of expressed PcGH61D

Ammonium sulphate was added to 150 mL of *P. pastoris* culture supernatant to a final concentration of 1 M, and the protein solution was applied to a Phenyl-Sepharose column (ϕ16 x 100 mm, GE Healthcare UK Ltd., Buckinghamshire, UK). Protein bound to the column was eluted by applying a linear reverse gradient of 1 to 0 M ammonium sulphate in 20 mM sodium acetate buffer pH 5.0. Fractions containing *Pc*GH61D, as monitored by SDS-PAGE analysis, were pooled and incubated with 0.5 µg of EndoH for 24 hours at 35°C. After buffer exchange to 20 mM Tris-HCl buffer, pH 8.0, containing 50 mM sodium chloride and 2 mM calcium chloride, using VIVAspin 20 (GE Healthcare UK Ltd., Buckinghamshire, UK). Approximately 10 mL of 1 mg/mL protein solution was incubated with 10 ng of enterokinase, light chain, at 30°C for 24 hours. The enterokinase treated protein solution was applied to a source30Q column (ϕ16 x 90 mm) equilibrated with 20 mM Tris-HCl buffer pH 8.0. Proteins were eluted from the column with a linear gradient from 0 to 0.25 M sodium chloride in the same buffer. This procedure yielded two peaks containing a 27 kDa and a 25 kDa protein, respectively. Fractions belonging to the same peak were diluted to 20 mM sodium acetate buffer pH 5.0 and concentrated to 1 mg/mL using VIVAspin 20 tubes (GE Healthcare UK Ltd., Buckinghamshire, UK).

### Sequence alignment and model building of PcGH61D

The Expresso program at the T-COFFEE Multiple Sequence Alignment Server [26] and ESPript [27] were used to prepare the structure based sequence alignment of *Pc*GH61D (residue 1 to 217 of the mature sequence starting from His 1) with the two GH61s with known crystal structures; GH61E from *Thielavia terrestris* (*Tt*GH61E; [15]; PDB code 3EII; (residue 1 to 208)) and Cel61B from *Hypocrea jecorina* (*Hj*Cel61B; [14]; PDB code 2VTC; residue 1 to 230). For comparison, the *Ta*GH61A sequence was included in the alignment.

A homology model of *Pc*GH61D was built using the SWISS-MODEL workspace [28], using the crystal structure of *Tt*GH61E (PDB code: 3EII) as template. *Pc*GH61D has 41% amino acid sequence identity with *Tt*GH61E. Structural comparisons were carried out with MacPyMOL (The PyMOL Molecular Graphics System, Version 1.3, Schrödinger, LLC, USA).

### Cellulosic substrates

Avicel PH-101 was obtained from Fluka analytical (Sigma Aldrich, St. Louis, USA). Cellulose nanofibrils were produced as described in [29], and were a kind gift from Prof. Dimitris Argyropoulos, NC State university. Phosphoric acid swollen cellulose (PASC) was prepared from Avicel using the method described by Wood [30]. Reduced PASC cellulose was prepared as follows: 2 mL of a 2% PASC suspension were centrifuged for 3 minutes at 14800 rpm, the supernatant was removed and the pellet was resuspended in 1 mL MilliQ H_2_O. After centrifugation (3 min, 21000 x g), the pellet was re-suspended in 4 mL 12.5 mM NaOH after which 25 mg NaBH_4_ was added and the tube was left at ambient temperature over night with occasional stirring. The reaction was quenched by neutralizing with 100 µL glacial acetic acid, followed by centrifugation as described above. The pellet was washed 4 times with MilliQ H_2_O and finally re-suspended in MilliQ H_2_O to obtain a 2% solution of reduced PASC.

### Functional studies

Various types of functional assays were conducted as described in the Results section, using chromatography and mass spectrometry for product identification as described in detail by Vaaje-Kolstad *et al*. and Forsberg *et al*. [7], [16]. Depending on the type of reactions, reaction products were detected by MALDI-TOF MS (Matrix assisted laser desorption ionisation time-of-flight mass spectrometry) as described by Vaaje-Kolstad *et al*. [16], and/or by High-Performance Anion-Exchange Chromatography (HPAEC) to detect native and oxidized cello-oligosaccharides, as described by Forsberg *et al*. [7], and/or by measuring release of cellotriose, cellobiose and glucose, as described in Forsberg *et al*. [7]. The *Pc*GH61D-containing chromatographic fraction used contained purified correctly processed *Pc*GH61D. The cellulase mixture used for synergy experiments was Celluclast 1.5 L (Novozymes, Copenhagen, Denmark).

Standard reaction mixtures contained the cellulose substrate (Avicel or filter paper 10 mg/mL; phosphoric acid swollen cellulose (PASC) or nano fibrillated cellulose 1mg/mL), 4–40 µg/mL *Pc*GH61D, 50 mM MES buffer pH 6.6, and 1.7 mM reduced glutathion, and reactions were incubated at 50°C with 900 rpm vertical shaking in an Eppendorf Thermo mixer. For the synergy experiments the enzyme concentrations were 56 µg/mL *Pc*GH61D and 1.3 µg/mL Celluclast 1.5 L (Novozymes, Copenhagen, Denmark). In experiments were MALDI-TOF analyses were applied for product analysis, the buffer was 25 mM Tris-HCl pH 6.5 and the reductant was 1 mM ascorbic acid (instead of reduced glutathione). Samples were taken at different time points. To obtain reproducible sampling condensed water from the upper part of tube was by spun down, and the sample was then collected after first mixing the contents of the tube with the pipette. The samples were centrifuged at 21000 x g for 3 minutes. Supernatants containing soluble oxidized oligosaccharides were collected and immediately applied to HPLC or MALDI-TOF analyses. The auto-sampler of the HPLC was kept at 4°C. Synergy experiments were run in triplicates. MALDI-TOF/MS spectra (see Vaaje-Kolstad *et al*. [16] for conditions) were acquired from averaging 250 arbitrary shots on each spot.

## Results

### Cloning, expression and purification of PcGH61D

In the two-dimensional gel electrophoresis analysis of *P. chrysosporium* culture filtrates, one protein spot at approximately 25 kDa molecular weight and pI of 4.8 showed much higher intensity when xylan had been added to the cellulose-containing medium. The N-terminal amino acid sequence of this protein was determined to be XYTFPDFIEPS. A BLAST search against the *P. chrysosporium* genome database revealed that this sequence did match with a *P. chrysosporium* gene model with protein ID 4691 (scaffold_7:1591640-1592893 in *P. chrysosporium* v. 2.0 genome database (http://genome.jgi-psf.org/Phchr1/Phchr1.home.html). Based on the annotated sequence, the corresponding cDNA was cloned and sequenced, and the correct prediction of the gene model with 10 introns was confirmed. The cloned cDNA consist of 708 bp, including an open reading frame encoding a 18-amino acid signal peptide at the N-terminal region followed by a 217-amino acid mature protein with a predicted mass of 23.5 kDa, and a theoretic pI value of 4.54. The protein coded for by the cDNA has two putative N-glycosylation sites and it consists of a single GH61 catalytic module that shows significant sequence similarity with previously characterized GH61 enzymes (22–41% identity). It has no CBM1 in contrast to the previously reported *Pc*Cel61A and B from *P. chrysosporium* that do, but in similarity with *Pc*Cel61C that does not [8], [18], [31]. This novel *P. chrysosporium* protein was designated *Pc*GH61D.

**Figure 1:**
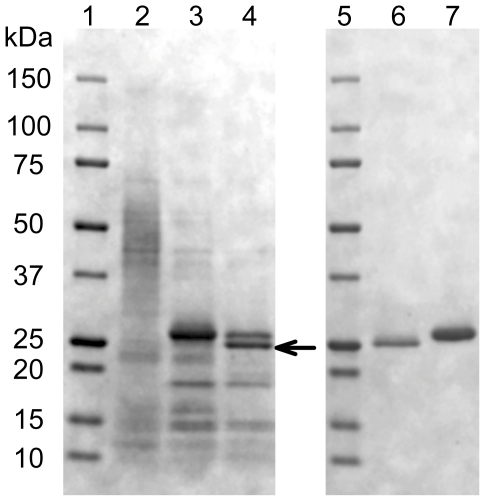
SDS-PAGE analysis of recombinantly expressed *Pc*GH61D. Lanes: 1 & 5, marker (Precision Plus Protein™ Dual Color Standards; BioRad); lane 2, culture medium of the *P. pastoris* strain; lane 3, EndoH treated culture medium; lane 4, EndoH treated culture medium after enterokinase treatment. Lane 6 & 7, protein bands with Mw of 25 and 27 kDa, respectively. The protein band used for N-terminal sequencing is indicated by an arrow.

**Figure 2:**
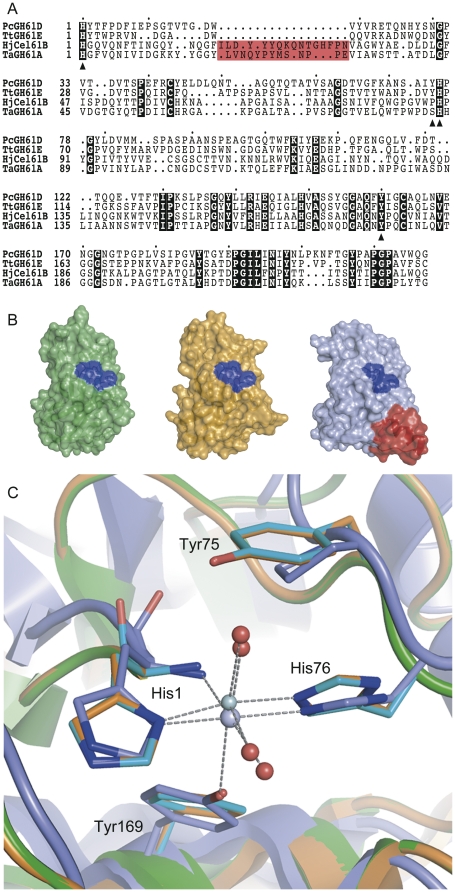
Sequence and structural analysis. Panel **A** shows a structure-based sequence alignment of *Pc*GH61D and GH61A from *Thermoascus aurantiacus* with GH61s with known crystal structures, *Tt*GH61E and *Hj*Cel61B. Residues in the metal ion-binding site of the proteins are indicated by triangles in the sequence alignment. The position of a large insertion in the *Hj*Cel61B structure and corresponding region in *Ta*GH61A is marked with a red box. Panel **B** shows surface representations of *Tt*GH61E (left), *Pc*GH61D (middle), and *Hj*Cel61B (right). The metal ion binding sites are coloured blue. The red surface in *Hj*Cel61B corresponds to the red box in panel A. Panel **C** shows a superposition of the metal binding sites of *Tt*GH61E (PDB code: 3EII, coloured green), *Hj*Cel61B (PDB code: 2VTC, coloured light-blue), and the *Pc*GH61D homology model (coloured orange). The light blue and green spheres indicate the nickel ion found bound in the *Hj*Cel61B structure and the zinc ion bound in the *Tt*GH61E structure, respectively. Red spheres indicate water molecules in the two structures *Hj*Cel61B and *Tt*GH61E. Grey dashed lines indicate interactions with the bound metals.

*Pc*GH61D was heterologously expressed in *P. pastoris* using a strategy where the key element was to generate an enterokinase cleavage site that would allow correct processing of the secreted protein, so that the catalytically important N-terminus of the mature recombinant protein would be the same as in the natural protein (i.e. starting with His1; see Materials and Methods for details). Figure 1 shows that the *P. pastoris* expressed *Pc*GH61D protein appeared as a smeary 40 to 50 kDa protein band on a SDS-PAGE gel (lane 2). However, after deglycosylation with EndoH a clear major band appeared at approximately 27 kDa (lane 3). Treatment with enterokinase led to formation of a 25 kDa protein (lane 4), which was shown by amino acid sequencing to have the correct N-terminal sequence, XYTFPDFI. Note that this is the same N-terminal sequence as found for the protein produced from natural sources [10], and that the first X must be a histidine (Fig. 2A). The 25 kDa and 27 kDa proteins could be separated into pure fractions of each by ion exchange chromatography and their purity was confirmed by SDS.PAGE (lanes 6 & 7). The protein shown in lane 7 is not active (data not shown), most likely due to incomplete processing of the N-terminus.

### Structure based sequence alignment and homology modeling of PcGH61D

Figure 2 shows a structure based sequence alignment of *Pc*GH61D with the two GH61 proteins for which the structure is known (*Tt*GH61E and *Hj*Cel61B), as well as a structural comparison based on the two crystal structures and a homology model of *Pc*GH61D. Figure 2C shows a close-up of the catalytic center highlighting four conserved residues His1, Tyr75, His76, and Tyr160 of *Pc*GH61D. Three of these four residues are highly conserved in family GH61 proteins [14] but Tyr75 is less conserved. This residue is substituted by a proline in *Hj*Cel61B and also in *Ta*GH61A recently described by Langston *et al*. [17], the sequence of which has been included in Figure 2A.

### Product analysis of PcGH61D

**Figure 3:**
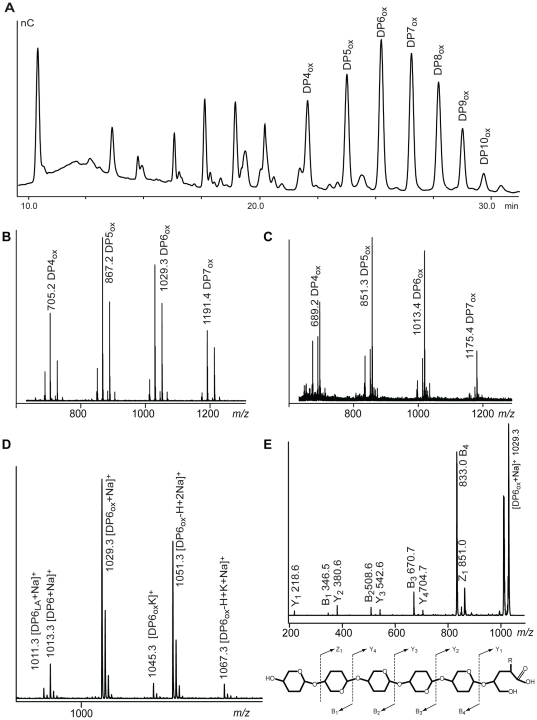
Products generated from cellulose by *Pc*GH61D. Panel **A** shows a typical HPAEC chromatogram of products obtained upon incubation of 0.1% PASC with 40 µg/mL *Pc*GH61D in 25mM Tris (not MES which is not good for MALDI) pH 6.5, 1mM ascorbic acid (not reduced glutathione which is not good for MALDI), overnight at 50°C. The chromatogram shows a range of oxidized oligosaccharides (DP 4-10) as well as native oligosaccharides (for information on chromatographic standards, see Forsberg *et al*. [7]). Panel **B** shows the MALDI spectrum of the same sample as in Panel **A** focusing on the most prominent masses in the spectrum. All ion clusters for oxidized products had a similar distribution, the most abundant peak being the Na-adduct of the aldonic acid, which is annotated with DPn_ox_ and its *m/z*. Panel **C** displays the MALDI-TOF-mass spectrum of the same sample as in panel **B** after saturation with lithium (20 mM) to obtain lithium adducts only. This was done to eliminate the possibility that the compound annotated as the aldonic acid in fact was a K-adduct of the native oligosaccharide (which would give the same mass as the Na-adduct of the oxidized oligosaccharide). Indeed Li-adducts ([M+Li]) and the corresponding lithium salt of the lithium adduct [M+2Li-H]) occur in pairs throughout the spectrum replacing completely the more complex cluster of Na- and K-adducts/salts (*m/z* -16 relative to the sodium adducts of panel **B**; only the lithium adducts of the aldonic acids are annotated). This confirms the presence of aldonic acids. For further clarification a detailed view of the ion cluster for DP6_ox_ (Glc_5_GlcA) from Panel **B** is shown in Panel **D**. The cluster contains the Na-adducts of the gluconolacton (DP6_La_, *m/z* 1011), cellohexaose (DP6, *m/z* 1013) and oxidized cellohexaose (DP6_ox_; *m/z* 1029). In addition, the spectrum shows the Na-salt of the Na-adduct of DP6_ox_ (*m/z* 1051), the K-adduct of DP6_ox_ (*m/z* 1045) and the Na-salt of the K-adduct of DP6_ox_ (*m/z* 1067). To verify the presence and position of the acid group, MS^2^ experiments were done for several ions. Panel **E** displays fragments obtained for DP6_ox_ (*m/z* 1029) with fragment ions named according to the Domon and Costello nomenclature [34]. The spectrum corresponds to that of a cello-oligosaccharide with an aldonic acid in the reducing end, and repeating hexose units towards the non-reducing end. The nomenclature used for products throughout this report is: DPn, cello-oligosaccharide with n glucose residues; DPn_ox_, cello-oligosaccharides with n-1 glucose residues + one gluconic acid [Glc_(n-1)_GlcA]; DPn_LA_, cello-oligosaccharides with n-1 glucose residues + one gluconolactone [Glc_(n-1)_GlcLA].

Figure 3 shows analyses of products released from cellulose upon incubation with *Pc*GH61D in the presence of a reducing agent. The data show that *Pc*GH61D releases both native and oxidized cello-oligosaccharides, similar to what has previously been observed for the CBM33 CelS2 [7]. The release of minor fractions of native cello-oligosaccharides is most likely due to the fact that polysaccharide chains have been cleaved close to their original reducing ends, as discussed in more detail below. Mass spectrometry analysis of the products revealed masses and adduct clusters that are typical for aldonic acids (Figure 3B-D). Furthermore, MS/MS fragmentation analysis of major ions (Figure 3E) showed that the end opposite to the non-reducing end carries the extra mass introduced by the oxidation. Minor peaks with masses possibly corresponding to the lactone form were also detected in the mass spectra (Figure 3D).

Several control experiments were done. Firstly, we were not able to detect any oxidized products when incubating *Pc*GH61D with soluble oligosaccharides (DP2-5), which is in accordance with the previous observation that CBM33s are not active on soluble chito- or cello-oligosaccharides [7], [16]. Secondly, incubation of 0.1% PASC in the presence of 1mM ascorbic acid and copper (II) sulfate at pH 5, which could lead to cellulose cleavage by enzyme-independent oxidative reactions, did not yield detectable levels of the products found in the reactions with *Pc*GH61D.

**Figure 4:**
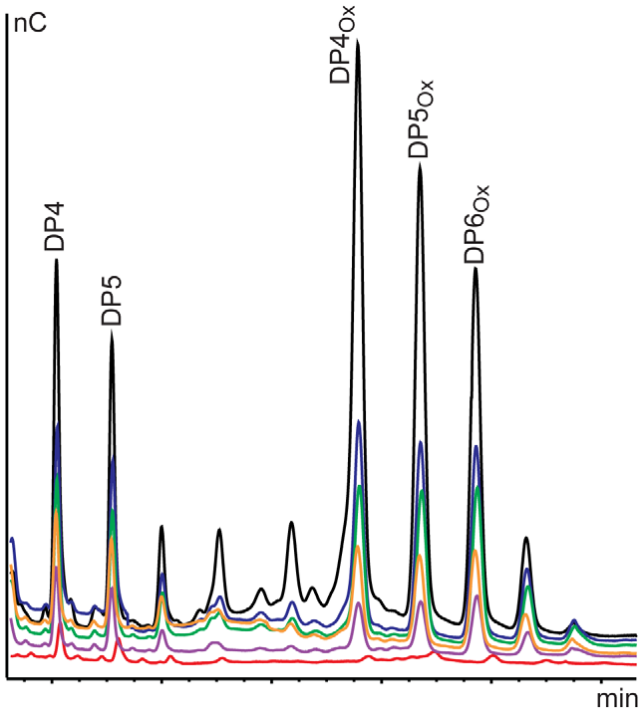
Effect of reductant concentration on *Pc*GH61D activity. The figure shows overlayed HPAEC chromatograms showing that release of oligosaccharides from cellulose by *Pc*GH61D increases with increasing ascorbic acid concentration. *Pc*GH61D (4 µg/mL) was incubated with 10 mg/mL Avicel in 50 mM MES buffer pH 6.6 containing different concentrations of ascorbic acid. The reactions were incubated for 24 hours at 50°C with vertical agitation at 900 rpm after which products were analysed. The ascorbic acid concentrations were 0 mM (red), 0.8 mM (magenta), 1.6 mM (orange), 2.0 mM (green), 2.4 mM (blue) and 4.8 mM (black).

By analogy to observations made for chitin-active CBM33s, the presence of reducing agents (i.e. external electron donors) was expected to have a beneficial effect on the cellulose-degrading activity of *Pc*GH61D. This is confirmed by Figure 4, which shows a clear dose-response effect of ascorbic acid on product formation. Other reductants such as reduced glutathione and gallic acid used at concentrations in the 1 mM range also stimulated *Pc*GH61D activity, approximately to the same level as ascorbic acid. Without addition of reductants the products of *Pc*GH61D was hardly detectable (Figure 4).

**Figure 5:**
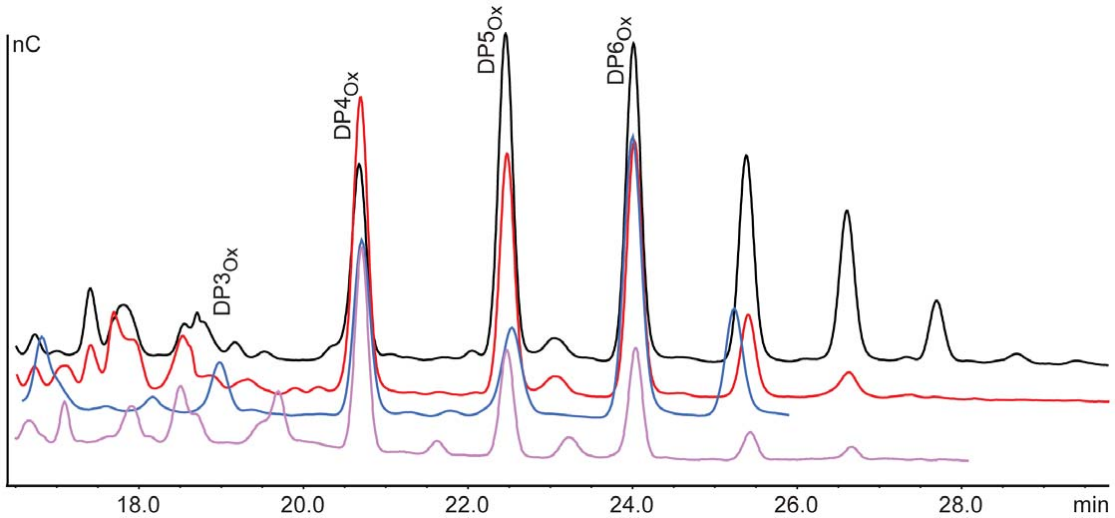
Effect of substrate and type of enzyme on product profile. The figure shows superposition of chromatograms as in the top panel of figure 3 for: PASC + *Pc*GH61D (black), Avicel + *Pc*GH61D (red), Cellulose nanofibrils + *Pc*GH61D (magenta), and Avicel + CelS2 (blue). Note the alternating intensities in the latter sample. All reactions were run in the presence of 1 mM ascorbic acid and at pH 6.5. Since these HPLC analyses were run over an extended time period the original chromatograms showed slight variations in elution times; this has been manually changed to obtain an optimum superposition. Standards were always run as part of each series of HPAEC experiments to secure correct peak annotation. Nb. Experiments with filter paper, which has a much higher DP (estimated to be ∼2000 pp. [35], did not yield detectable amounts of soluble products. However, when a cellulase was added, short oxidized products were detected in chromatograms that looked approximately as the chromatogram obtained in the synergy experiment including GH61 in Figure 8B.

Figure 5 shows that similar product profiles were obtained when incubating *Pc*GH61D with different types of cellulosic substrates, including nanofibrillated cellulose Avicel and PASC. For comparison, a product profile obtained with the cellulolytic CBM33 CelS2 is also shown. While the product profiles generally are quite similar, they do show subtle differences with respect to the length distributions of the released products. Notably, CelS2 yields products that are predominantly even-numbered, whereas *Pc*GH61D does not (see below for further discussion).

### Effect of metal ions

**Figure 6:**
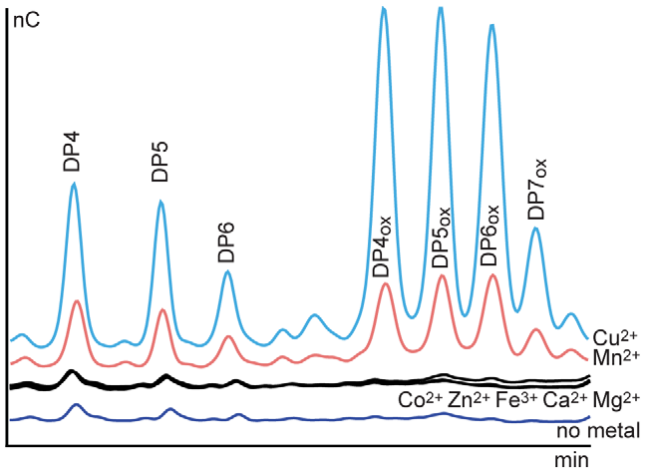
Metal dependency of *Pc*GH61D. 0.8 mg/mL *Pc*GH61D was incubated with 400 µM EDTA for 3 hours at 20°C. This EDTA treated *Pc*GH61D (final concentration 40 µg/mL) was incubated with 10 mg/mL Avicel in 50 mM MES buffer pH 6.6 containing 1.7 mM reduced glutathione and one of 7 different metal ions (10 µM; Mg^2+^, Fe^3+^, Zn^2+^, Co^2+^, Ca^2+^, Cu^2+^, Mn^2+^). The final concentration of EDTA in the reaction mixtures was 20 µM. The superimposed HPAEC chromatograms show products released after 20 hours of incubation at 50°C. Incubation of Avicel without addition of *Pc*GH61D under these same conditions led to release of small amounts of native oligomeric products from the substrate and yielded a chromatogram similar to that labelled “no metal”.

GH family 61 proteins have a metal binding site and it is known that metals are necessary for activity [15]. In the experiments described above it was not necessary to add extra metals to the reaction mix (addition did not lead to higher activity), indicating that sufficient amount of metals were already present in the purified protein and/or in the substrates. In an attempt to identify which is the preferred metal for GH61, a stock solution of enzyme was treated by ethylenediaminetetraacetic acid (EDTA), and different metals were tested to reactivate the protein. Figure 6 clearly shows that under the conditions used in this experiment, including use of very low metal concentrations, only copper and manganese were able to reactivate *Pc*GH61D (at a 10 µM metal concentration in the presence of 20 µM EDTA). At 5 µM metal concentration only copper could activate the enzyme (data not shown).

### Action of PcGH61D on reduced cellulose

**Figure 7:**
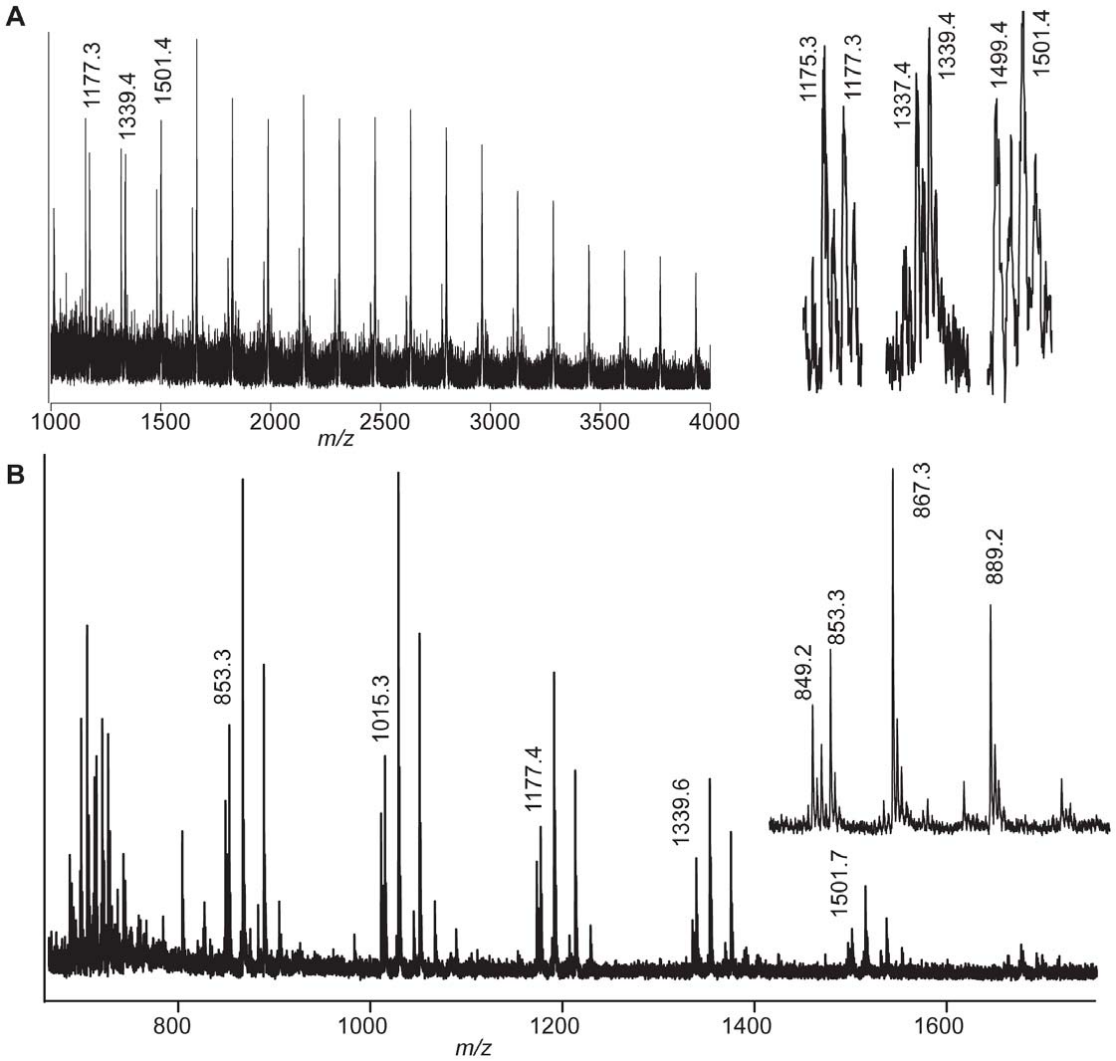
Effect of *Pc*GH61D on reduced cellulose. Panel **A** displays a MALDI-TOF-MS spectrum of reduced PASC, showing peak series differing by *m/z* 162-repeats, typical for hexose oligosaccharides, up to *m/z* 4000. Note that the reduction is not complete; the close-up to the right shows that the 1177, 1339 and 1501 peaks, corresponding the reduced heptamer, octamer and nonamer, respectively, are accompanied by a peak corresponding to the native oligosaccharides with *m/z* -2 (two protons less). The reduced PASC (0.1%) was incubated with 40 µg/mL *Pc*GH61D in 20mM Tris buffer pH 6.5, 1.0 mM ascorbic acid, at 50°C, and samples were taken at 90 minutes, 4 and 20 hours. The MALDI-TOF-mass spectrum of the 4 hour sample (Panel **B**) and clearly shows reduced products (their *m/z* values are indicated). The close-up to the right shows the various pentameric products (sodium adducts are marked): the DP5 lactone, *m/z* = 849; DP5, *m/z* = 851 (minor amount, not labelled); reduced DP5, *m/z* = 853.3; DP5_ox_, *m/z* = 867.3; sodium salt of DP5_ox_ (-H^+^, +Na^+^), *m/z* = 889.2).

GH family 61 proteins have till now been claimed to possess a weak endoglucanase activity, based on the release of low amounts of cello-oligosaccharides from certain substrates. In order to investigate whether native cello-oligosaccharides released from cellulose by GH61 have reducing ends that were already present in the original substrate and thus not a result of GH61 hydrolytic activity, a reduced cellulose substrate was prepared in which the reducing ends are converted to glucitols. Figure 7 shows that the major products observed upon degradation of this substrate by *Pc*GH61D were reduced and oxidized cello-oligosaccharides, whereas the amounts of released native cello-oligosaccharides were much lower than observed with normal cellulose. Interestingly, the amounts of oxidized oligosaccharides compared to glucitol oligosaccharides, as estimated from the mass spectra, increased over time (3:1 after 4 h; 13:1 after 20 h). Furthermore, the native:reduced ratio increased over time (not shown). The fact that reduced products became less prominent in the later phase of the reaction with reduced PASC may be taken to indicate that the enzyme penetrates the substrate where the number of existing chain ends and/or the degree of reduction of these chain ends are likely to be lower. Taking into account that the reduction of the substrate was incomplete (Figure 7A), these data clearly show that at least the large majority and, possibly, all of the native products observed when incubating PASC with *Pc*GH61D have reducing ends that were already present in the substrate and that have thus not been generated by the enzyme.

### Synergy with cellulases

**Figure 8:**
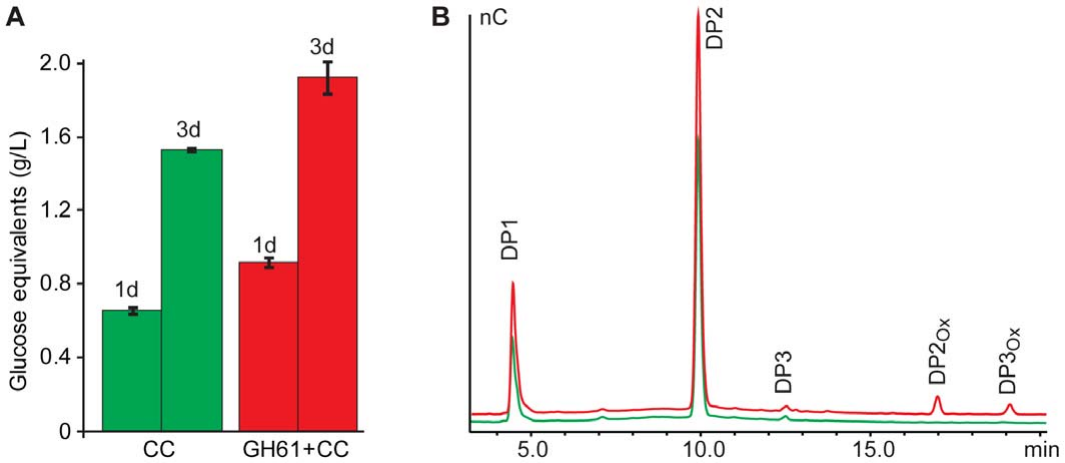
Synergy between *Pc*GH61D and Celluclast. Panel **A** shows glucose release from 10 mg/mL Avicel by Celluclast (CC) after 1 day (1d) and 3 days (3d) for reactions with (red bars) or without *Pc*GH61D (green bars). Reactions incubated with *Pc*GH61D alone did not release detectable amounts of glucose or cellobiose. Panel **B** shows typical chromatograms of the soluble sugars obtained in these reactions (1 day) with accumulation of Glc and Glc_2_ and some Glc_3_ (green, reaction without *Pc*GH61D; red, reaction with *Pc*GH61D). In the case of reactions with *Pc*GH61D, the oxidized oligosaccharides GlcGlcA (DP2_ox_) and Glc_2_GlcA (DP3_ox_) were also observed. In the reactions without *Pc*GH61D, BSA was added to keep the protein concentration constant in all reactions. See Materials & Methods section for conditions.

To check for synergistic effects with cellulases, cellulose was degraded by Celluclast in the presence or absence of *Pc*GH61D. Figure 8 shows a synergistic effect of adding GH61 to the reaction mixture. Figure 6B shows that the main products produced by Celluclast are glucose and cellobiose. However, the presence of GH61 also leads to accumulation of oxidized dimers and trimers. These oxidized products are not included as glucose equivalents in Panel A, meaning that the synergistic effect shown by this figure is somewhat underestimated.

## Discussion

Characterization of GH61 proteins was described for the first time in 1992 [32], but their function has remained enigmatic [15] until very recently ([16], [33], and this paper). They were originally thought to exhibit weak endoglucanase activity based on the detection of low levels of newly formed reducing end upon the incubation of the protein with cellulose [13]. The data described here show that *Pc*GH61D does cleave cellulose but that this generally does not lead to generation of new reducing ends. *Pc*GH61D-catalyzed oxidative cleavage of cellulose close to existing reducing ends will release soluble native cello-oligosaccharides. This was indeed observed and might explain why low endoglucanase activities have been detected in some previous studies. It should be noted though that although the experiments with reduced cellulose show that the large majority of released native cello-oligosaccharides have reducing-ends that were already present in the substrate, we cannot exclude that *Pc*GH61D has a side-activity that entails hydrolysis without oxidation.

A potential boosting effect of GH61 proteins on cellulose activity was reported in 2007 [1] and underpinned in a later study by Harris *et al*. [15]. The discovery of the chitin-cleaving activity of the structurally homologous CBM33 proteins [16] provided a possible explanation for this GH61 activity. Indeed, we show here that *Pc*GH61D has a similar effect on cellulose as the chitin-active CBM 33 CBP21 has on chitin and as the cellulose-active CBM33 CelS2 has on cellulose [7]. *Pc*GH61D cleaves cellulose at the glycosidic bond, leaving one of the new chain ends oxidized to a lactone, which subsequently is spontaneously converted to an aldonic acid in solution. The enzyme is not active on soluble cello-oligosaccharides and its flat active site-containing surface, devoid of potential substrate-binding grooves and pockets [14], [15], suggests that it is optimized for interacting with ordered substrate surfaces such as they occur in crystalline and otherwise well-ordered substrates. This is analogous to what has been claimed, and to a large extent proven [16], for CBM33s.

The activity of both CBM33s and GH61s is increased in the presence of external electron donors, be it chemical reductants such as ascorbic acid (see above) or electron generating enzyme systems such as CDH [17]. It should be noted though that the few studies now available in the literature show differences between the systems studied. The original work on chitin-active CBP21 showed dramatic effects both in terms of the effect of adding reductants and the boosting effect on chitinase activity [16]. For the cellulolytic CBM33 and GH61 enzymes studied so far, these effects are less dramatic and generally in the same range as the effects described here for *Pc*GH61D [7], [15], [17]. The product profiles in Figure 5 reveal differences between cellulolytic CBM33s and GH61s and between substrates. For example, whereas the products generated from Avicel by CelS2 (a CBM33) show a dominance of even-numbered products, as previously seen for CBP21 acting an chitin (see Vaaje-Kolstad *et al*., 2010 for further discussion), the products released by *Pc*GH61D from the same substrate do not show this periodicity. Figure 5 also shows that chromatographic product profiles generated by *Pc*GH61D differ slightly for the different substrates. It is conceivable that these differences reflect different binding modes of the enzymes; for example, the enzymes may vary in terms of their ability to act on substrate regions of varying crystallinity or they may bind to different faces of the crystalline material. Interestingly, the recent study by Langston *et al*. [17] indicated that *Ta*GH61A, which shows some interesting sequence differences with *Pc*GH61D (Figure 2A) may in fact have a slightly different oxidative mechanism since several products were detected in addition to aldonic acids. Further work is needed to unravel the causes and implications of the possible differences between members of the GH61 and CBM33 families.

Another issue that so far has remained partly unresolved is the dependency of both CBM33s and GH61s on bivalent metals. Initially, it was reported for both types of enzymes that they can work with a wide variety for bivalent metal ions, including several that are not redox-active [15]. It is likely that these remarkable observations were partly misinterpreted due to the fact that the concentrations of added metals were too high, thus releasing already present metals from binding sites through displacement. It would in fact be quite remarkable if the activity of these redox enzymes did not depend on the presence of a redox metal. Our present data indicate quite clearly that *Pc*GH61D is a copper oxidase, and that it can use manganese in the absence of copper. Interestingly, our own (partly unpublished) observations on the metal-dependency of CBM33s have so far not yielded equally clear results.

When this paper was about ready for submission, a report by Quinlan *et al*. appeared online that describes an in-depth study of *Ta*GH61A and demonstrates that this is an oxidative enzyme that cleaves cellulose [33]. *Ta*GH61A resembles *Pc*GH61D (and cellulose-active CBM33s; [7]) in that the enzyme produces aldonic acids and works better in the presence of redox-active cofactors such as ascorbate. The study by Quinlan *et al*. also provides evidence for *Ta*GH61A being a copper oxidase. Most interestingly, this very recent study also confirmed differences between *Pc*GH61D and *Ta*GH61A, since the latter enzyme was found to produce additional oxidized species that we have not observed for *Pc*GH61D. As noted above and in Figure 2, the sequences of the two enzymes show some interesting differences that could underlie functional differences.

The recent findings on CBM33 and GH61 proteins add a completely new dimension to the classical concept of cellulose degradation by endo- and exo-acting cellulases. Clearly, nature has developed additional oxidative enzyme systems for tackling this recalcitrant substrate that may be specifically tailored for acting on the least accessible regions. It is important to note the abundance of GH61 in fungal genomes and the (not quite as large) abundance of CBM33s in some bacterial genomes. It seems quite likely that additional substrate specificities will be discovered for these proteins. For example, GH61s acting on chitin are likely to exist, as are GH61s acting on certain xylans, galactomannans, or heteropolymeric polysaccharide materials. The data presented in Figure 5 and discussed above suggest functional differences between cellulolytic GH61s/CBM33s and it is thus conceivable that synergistic effects may be observed when combining several of these proteins working on the same substrate. We envisage that more knowledge on these GH61 and CBM33 enzymes will contribute considerably to our general understanding of enzymatic biomass conversion and open up new avenues towards efficient industrial biomass processing.
